# Personalized Pressure Conditions and Calibration for a Predictive Computational Model of Coronary and Myocardial Blood Flow

**DOI:** 10.1007/s10439-024-03453-9

**Published:** 2024-02-09

**Authors:** Giovanni Montino Pelagi, Andrea Baggiano, Francesco Regazzoni, Laura Fusini, Marco Alì, Gianluca Pontone, Giovanni Valbusa, Christian Vergara

**Affiliations:** 1 LABS, Dipartimento di Chimica, Materiali e Ingegneria Chimica, Politecnico di Milano, 20133 Milan, Italy; 2 Perioperative Cardiology and Cardiovascular Imaging Department, Centro Cardiologico Monzino IRCCS, Via Carlo Parea 4, 20138 Milan, Italy; 3 Department of Clinical Sciences and Community Health, University of Milan, Milan, Italy; 4 MOX, Dipartimento di Matematica, Politecnico di Milano, Piazza Leonardo da Vinci 32, 20133 Milan, Italy; 5 Bracco Imaging S.p.A., Via Caduti di Marcinelle 13, 20134 Milan, Italy; 6 Department of Diagnostic Imaging and Stereotactic Radiosurgery, Centro Diagnostico Italiano S.p.A., Via Saint Bon 20, 20147 Milan, Italy; 7 Department of Biomedical, Surgical and Dental Sciences, University of Milan, 20134 Milan, Italy; 8 Department of Electronics, Information and Biomedical Engineering, Politecnico di Milano, 20133 Milan, Italy

**Keywords:** Coronary artery disease, Fractional flow reserve, Myocardial perfusion, Myocardial blood flow, Computational modeling, Coronary pressure

## Abstract

Predictive modeling of hyperemic coronary and myocardial blood flow (MBF) greatly supports diagnosis and prognostic stratification of patients suffering from coronary artery disease (CAD). In this work, we propose a novel strategy, using only readily available clinical data, to build personalized inlet conditions for coronary and MBF models and to achieve an effective calibration for their predictive application to real clinical cases. Experimental data are used to build personalized pressure waveforms at the aortic root, representative of the hyperemic state and adapted to surrogate the systolic contraction, to be used in computational fluid-dynamics analyses. Model calibration to simulate hyperemic flow is performed in a “blinded” way, not requiring any additional exam. Coronary and myocardial flow simulations are performed in eight patients with different clinical conditions to predict *FFR* and MBF. Realistic pressure waveforms are recovered for all the patients. Consistent pressure distribution, blood velocities in the large arteries, and distribution of MBF in the healthy myocardium are obtained. *FFR* results show great accuracy with a per-vessel sensitivity and specificity of 100% according to clinical threshold values. Mean MBF shows good agreement with values from stress-CTP, with lower values in patients with diagnosed perfusion defects. The proposed methodology allows us to quantitatively predict *FFR* and MBF, by the exclusive use of standard measures easily obtainable in a clinical context. This represents a fundamental step to avoid catheter-based exams and stress tests in CAD diagnosis.

## Introduction

Coronary artery disease (CAD) represents a widespread pathological condition responsible of the largest amount of deaths worldwide. Because the most critical cases require invasive surgical procedures (e.g. revascularitazion) with many risks associated [1], prognostic stratification of CAD is of paramount importance for the definition of optimal treatment options. Within this context, the assessment of cardiac perfusion through the quantification of Myocardial Blood Flow (MBF) at the cardiac tissue level is of crucial interest [2].

In current clinical practice, the gold standard is the invasive coronary angiography (ICA) together with the measurement of the fractional flow reserve (*FFR*) index, a widely used and reliable predictor of the hemodynamic impact of epicardial coronary lesions [3]. However, due to the invasiveness of the procedure and the need to induce a pharmacological stress condition in the patient (hyperemia), the prescription of such exam is recommended only when strictly necessary [4]. For this reason, there is great interest in enhancing the prognostic power of non-invasive exams, such as the coronary computed tomographic angiography at rest (cCTA). This technique allows for the detection and quantification of coronary lesions with great accuracy from an anatomical standpoint [2, 5], but it does not allow to assess the hemodynamic relevance of such lesions nor their impact on the MBF. The latter can be clinically assessed through a further CT scan in stress conditions (stress-CTP exam) with additional radiation exposure and the administration of a stressor agent.

Computational models of coronary blood flow (CBF) have been proposed as a supporting tool in prognostic stratification, performing for example a patient-specific functional analysis on top of the anatomical data extracted from cCTA images [6, 7]. The most prominent example is the HeartFlow®analysis [8], which relies on computational fluid dynamics simulations in the major coronary arteries to compute the FFR index in a non-invasive way, known as $FFR_{CT}$. The main challenges in the field are the prescription of accurate and personalized boundary conditions, which often require either direct clinical measures (that are usually not feasible in clinical practice) [9] or surrogate 0D models (introducing a large number of parameters that may be difficult to estimate) [8, 10], and the difficulties in evaluating lesion-specific effects on the MBF at the tissue level.

In our previous works, we proposed a multiscale framework for CBF simulations from the large arteries up to the microvasculature at the cardiac tissue level [11], in what follows referred to as *CBF-Perfusion simulations*, and its application to real clinical cases for MBF quantification [12]. The main limitation was that data from the stress-CTP exam were required for a successful calibration of the myocardial constitutive parameters and for the prescription of accurate inflow boundary conditions.

In this context, the present paper presents two major novelties with respect to previous publications:

1. The first aim of this work is to propose a new way to build an optimized inlet boundary condition in the form of a parametrized hyperemic pressure profile over time at the aortic root;
2. Secondly, we propose a new, “blinded” calibration procedure of the CBF-Perfusion model parameters, alternative to [12], so to avoid the use of stress-CTP data.

We applied these two new tools in hyperemic CBF-Perfusion simulations of eight patients, with the aim of quantitatively predicting *FFR* and *MBF*.

## Methods

In “Pressure Waveform Reconstruction” section we present the framework used to build the pressure curve, to be prescribed as inlet condition to the CBF-Perfusion model, starting from patient-specific data, whereas in “Adaptation to Non-contracting Computational Models” section we discuss how to adapt it to computational models that do not include the effects of cardiac contraction. In “Data-Driven Estimation of Hyperemic Data” section we present the data-driven parametrization of the pressure curve representative of the hyperemic state; finally, in “Coronary Blood Flow-Perfusion Model and Calibration and Quantities for Validation” sections we discuss the CBF-Perfusion computational model, the new “blinded” calibration strategy, and the benchmark quantities we used for validation.

### Pressure Waveform Reconstruction

The blood pressure waveform over the cardiac cycle at the aortic root ($P_{\text {ar}}$) shows a characteristic shape that is the result of different physiological processes, including systolic ejection, aortic valve mechanics and compliance of the aorta. This has been taken into account in the mathematical description of the pressure waveform by using a continuous, piecewise polynomial function of time where the various time phases are approximated by different polynomial functions inspired by the specific time evolution in that region. Notice that we did not enforce continuity of derivatives, thus allowing sharp variation of the pressure waveform. This, in our opinion, better represents physiology, since non-smooth variations have been reported by invasive pressure measures, e.g. in the case of aortic incisura [13]. These sharp variations could induce numerical artifacts or even instabilities that were prevented by using a small value of the time discretization parameter, which however was already a requirement accounted for numerical stability of the whole coupled problem.

**Fig. 1:**
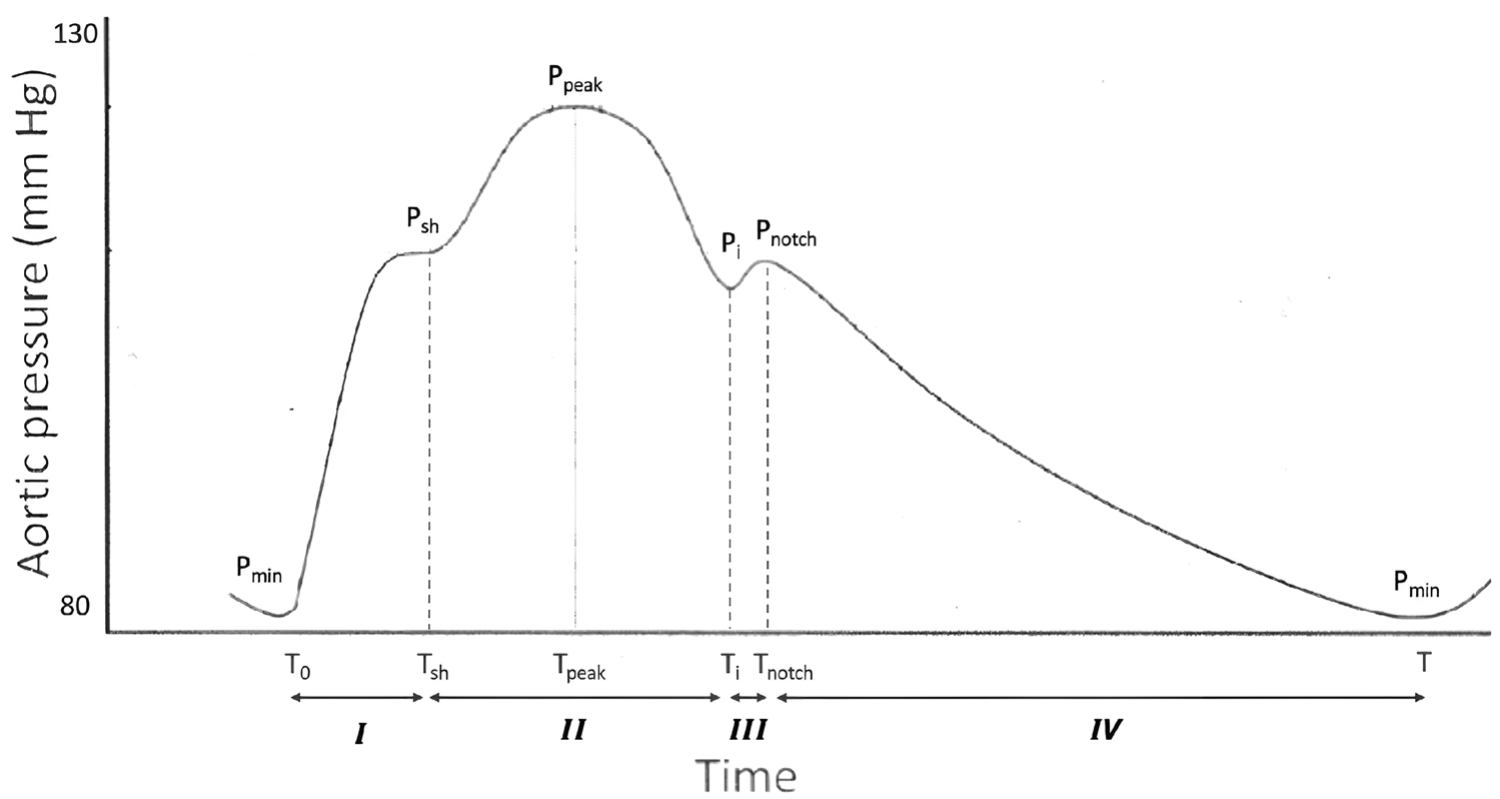
Characteristic pressure waveform at the aortic root with the proposed 4-phases subdivision, key time instants and corresponding pressure values

The target waveform is subdivided into 4 regions (I–IV, see Fig. 1) identified by characteristic time instants. These instants, their corresponding pressure values and the chosen polynomial approximation are summarized in Table 1.

**Table 1:** Time subdivision of the aortic pressure waveform including starting and end times, starting and end pressures and choice of the polynomial degree

Region	Time interval	Pressure interval	Polynomial
$P^I$—Syst. upstroke	$T_{0}$: initial time	$P_{min}$: end diastolic P	$3\mathrm{{rd}}$ order
	$T_{\text {sh}}$: syst. shoulder time	$P_{\text {sh}}$: syst. shoulder P	
$P^{II}$—Augmentation	$T_{\text {sh}}$: syst. shoulder time	$P_{\text {sh}}$: syst. shoulder P	$2\mathrm{{nd}}$ order
	$T_{i}$: incisura time	$P_{i}$: incisura P	
$P^{III}$—Dictrotic notch	$T_{i}$: incisura time	$P_{i}$: incisura P	$2\mathrm{{nd}}$ order
	$T_{\text {notch}}$: dicr. notch time	$P_{\text {notch}}$: dicr. notch P	
$P^{IV}$—Diastole	$T_{\text {notch}}$: dicr. notch time	$P_{\text {notch}}$: dicr. notch P	$1\mathrm{{st}}$ order
	*T*: end diastolic time	$P_{\text {min}}$: end diastolic P	

#### Computation of the Pressure Waveform Coefficients

In each of the time regions, the pressure waveform is therefore described by a polynomial whose coefficients depend on the patient and are computed as described in this subsection. The only requirement is the knowledge of $P_{\text {ar}}$ at the key time instants of Fig. 1, which can be achieved through computation based only on patient’s basic information and measures.

**Table 2:** Required information for aortic pressure parametrization. The pressure measures $P_{\text {sys}}$ and $P_{\text {dia}}$ represent systolic and diastolic pressure values as measured at the arm level (brachial artery) by standard clinical means

Input type	Input
Basic information	Age, sex
	Height, weight
Routine clinical measures	*HR* heart rate
	$P_{\text {sys}}$ brachial systolic pressure
	$P_{\text {dia}}$ brachial diastolic pressure
Imaging-derived	Left ventricular mass

**Table 3:** Parameters computed for a specific patient from the inputs in Table 2

Parameter	Physiological meaning	Computation
$P_{\text {min}}$	Aortic minimum diastolic pressure	$P_{\text {min}} = P_{\text {dia}}$ [14]
$T_{\text {sh}}$	Time to systolic shoulder	$T_{\text {sh}} = \frac{1}{3}T_{\text {notch}}$ [15]
$P_{\text {sh}}$	Systolic shoulder pressure	From $P_{\text {peak}}, \Delta P$ with Eq. (2)
$\Delta P$	Augmentation pressure (reflected P wave)	From metadata with Eq. (3) [16]
$T_{\text {peak}}$	Time instant of peak systolic pressure	$\frac{1}{2}T_{\text {notch}}$ [17]
$P_{\text {peak}}$	Aortic maximum systolic pressure	$P_{\text {peak}} = P_{\text {sys}} - 10 \, {\text {mmHg}}$ [14]
$T_{i}$	Incisura time (closing of aortic valve)	$T_{i} = T_{\text {notch}}-IVRT$
*IVRT*	Isovolumic relaxation time (interval)	Regression on data [18], Eqs. (6)–(8)
$P_{i}$	Aortic incisura pressure	Evaluating $P^{II}$ at $t = T_{i}$
$T_{\text {notch}}$	Dicrotic notch time (systolic duration)	Regression on data [19], Eq. (5)
$P_{\text {notch}}$	Dicrotic notch pressure	Regression on data [20], Eq. (10)
*IVCT*	Isovolumic contraction time	$IVCT = 0.05$ s [21]
*T*	Total heartbeat time	$T = \frac{60}{HR}$

Column 3 specifies whether the computation is taken directly from literature studies or if it is performed through a regression on experimental data

A list of the required inputs is reported in Table 2, whereas Table 3 reports the related parameters, alongside their physiological meaning and a general indication on how they were computed.

The parametrization of the four polynomials representing the pressure waveforms has been obtained as follows:

(I) *First systolic upstroke* ($0< t < T_{\text {sh}}$): obtained through the solution of:

1 $$\begin{aligned} {\left\{ \begin{array}{ll} P^I(t) = a_It^3 + b_It^2 +c_It + d_I, \\ P^I(0) = P_{\text {min}}, \\ P^I(T_{\text {sh}}) = P_{\text {sh}}, \\ \frac{\partial {P^I}}{\partial {t}}|_{t=T_{\text {sh}}} = k. \\ \end{array}\right. } \end{aligned}$$

In problem (1), $P_{\text {min}}$ is taken equal to $P_{\text {dia}}$ as suggested by previous findings [14]; $T_{\text {sh}}$ has been consistently found to occur at one third of $T_{\text {notch}}$ for individuals over 40 years old [15]. $P_{\text {sh}}$ can be expressed as:

2 $$\begin{aligned} P_{\text {sh}} = P_{\text {peak}} - \Delta P; \end{aligned}$$

where $\Delta P$ is called the augmentation pressure, computed as follows [16]:

3 $$\begin{aligned} \left. \begin{array}{l} Men: \, \Delta P/(P_{\text {peak}}-P_{\text {min}}) = \\ \qquad 79.70 + 0.63 \, age -0.002 \, age^2 -0.28 \, HR -0.39 \, height_{cm}, \\ Women: \, \Delta P/(P_{\text {peak}}-P_{\text {min}}) = \\ \qquad 56.28 + 0.90 \, age -0.005 \, age^2 -0.34 \, HR -0.24 \, height_{cm}; \\ \end{array}\right. \end{aligned}$$

Notice that, as known, $P_{\text {peak}}$ is smaller than $P_{\text {sys}}$ due to the increased stiffness of the distal arteries with respect to the aorta [14]. Therefore, we here set $P_{\text {peak}} = P_{\text {sys}} - 10 \, {\text {mmHg}}$ and $P_{\text {peak}} = P_{\text {sys}} - 8 \, {\text {mmHg}}$ for men and women, respectively [14]. Lastly, *k* in (1) is an empirical parameter used to improve the smoothness in the transition from time region 1 to region 2, and was set to $k = 75$ mmHg/s.

(II) *Augmentation region* ($T_{\text {sh}}< t < T_{i}$): obtained through the solution of:

4 $$\begin{aligned} {\left\{ \begin{array}{ll} P^{II}(t) = a_{II}(t - T_{\text {peak}})^2 + P_{\text {peak}}, \\ P^{II}(T_{\text {sh}}) = P_{\text {sh}}. \\ \end{array}\right. } \end{aligned}$$

$T_{\text {peak}}$ corresponds to the time of arrival of the reflected pressure wave and it was set equal to $T_{\text {notch}}/2$ [17]. The pressure curve in this time region can then be used to compute the pressure $P_{i}$ at the incisura time $T_{i}$, which is defined as:

$$\begin{aligned} T_{i} = T_{\text {notch}} - IVRT, \end{aligned}$$

where IVRT is the isovolumic relaxation time. Given that systole starts at $T_0 = 0$, $T_{\text {notch}}$ coincides with the duration of the systolic phase and, for its computation, we here use a $2{\text {nd}}$ order fitting we built on the experimental data from Bombardini et al. [19]. This method leads to the diastolic/systolic time ratio $R_{d-s}$, depending on the *HR* in bpm:

5 $$\begin{aligned} R_{d-s} = 2.537*10^{-4} \, (HR)^2 - 0.057 \, HR + 4.3, \end{aligned}$$

which can be used to easily compute both the systolic duration $T_{\text {notch}}$ and diastolic duration $T - T_{\text {notch}}$. Regarding the computation of IVRT, previous studies pointed out that this duration depends mainly on age and Left Ventricular Indexed Mass (LVIM) [18], so here we propose a double-regression method based on experimental data from Larrazet et al. [18], which include measures of IVRT both on a population of healthy controls of varying age (range 15–90 years) and on a mixed population including also patients affected by left ventricular hypertrophy (increased left ventricular mass) with a rather narrow age range (mean age $54 \pm 14$ years). The double-regression method we propose consists in the following steps:

1. Data from the healthy control group were used to build the linear regression $IVRT_{\text {normal}}$ vs age:
  6 $$IVRT_{{{\text{normal}}}} [{\text{ms}}] = 0.412\;age\;[{\text{y}}] + 47.882;$$
2. Data from the patients group were used to build the linear regression $IVRT^{54}(ms)$ vs LVIM, with the superscript “54” representing the mean age of the population and the LVIM was computed with the Du Bois formula using the subject height and weight:
  7 $$IVRT^{{54}} [{\text{ms}}] = 0.267\;LVIM[{\text{g/m}}^{{\text{2}}} ] + 51.45;$$
3. The regression as in point 2 is corrected accounting for age with a shifting factor $s = 0.412*(age-54)$, derived from the regression described in point 1. The final double regression reads:
  8 $$\begin{aligned} IVRT[ms] = 0.267 \, LVIM[g/m^2] + 51.45 + s. \end{aligned}$$

(III) *Dicrotic notch region* ($T_{i}< t < T_{\text {notch}}$): obtained through the solution of:

9 $$\begin{aligned} {\left\{ \begin{array}{ll} P^{III}(t) = a_{III}(t - T_{\text {notch}})^2 + P_{\text {notch}}, \\ P^{III}(T_{i}) = P^{II}(T_{i}). \\ \end{array}\right. } \end{aligned}$$

In problem (9), we computed $P_{\text {notch}}$ from a simplified mean pressure $P_{\text {mean}} = 0.5*(P_{\text {peak}} + P_{\text {min}})$ using a regression method we built on experimental data from Hèbert et al. [20]. The obtained regression line (R = 0.974) was:

10 $$P_{{{\text{notch}}}} [{\text{mmHg}}] = 1.1667\;P_{{{\text{mean}}}} [{\text{mmHg}}] - 12.629.$$

IV) *Diastole* ($T_{\text {notch}}< t < T$): obtained from the solution of:

11 $$\begin{aligned} {\left\{ \begin{array}{ll} P^{IV}(t) = a_{IV}t + b_{IV}, \\ P^{IV}(T_{\text {notch}}) = P_{\text {notch}}, \\ P^{IV}(T) = P_{\text {min}}. \end{array}\right. } \end{aligned}$$

Summarizing, the aortic pressure waveform $P_{\text {ar}}$ piecewise reconstructed with this method reads:

12 $$\begin{aligned} P_{\text {ar}}(t) = \left\{ \begin{array}{ll} P^I&{}t\in [0,T_{\text {sh}}]\\ P^{II}&{}t\in [T_{\text {sh}},T_i]\\ P^{III}&{}t\in [T_i,T_{\text {notch}}]\\ P^{IV}&{}t\in [T_{\text {notch}},T]\\ \end{array} \right. \end{aligned}$$

This pressure waveform can be used as an inlet boundary condition, prescribed at the coronary ostia, in any computational model of coronary circulation. However, models that do not include the effects of cardiac contraction require a special treatment that is discussed in the following section.

We notice that continuity of the proposed pressure waveform has been guaranteed by the matching conditions (1)_3_,   (3)_2_,   (9)_2_,   (11)_2_.

### Adaptation to Non-contracting Computational Models

One of the disadvantages of using a prescribed pressure inlet condition in coronary flow models (instead of a more standard prescription of the inflow over time) is that, if the effects of cardiac contraction are neglected, the higher systolic pressure produces higher flows in systole rather than diastole, which is not in accordance with the physiology of coronary circulation [22]. As experimental studies [23] pointed out, the pressure buildup inside the ventricular chamber, resulting from systolic contraction, has a major limiting effect on systolic coronary flow due to the compressive force on the microvasculature.

To take these effects into account without including a very complex and computationally expensive contraction model, we propose to correct the inlet boundary condition (12) as follows:

13 $$\begin{aligned} P_{\text {eff}} = P_{\text {ar}} - P^*_{\text {LV}}, \end{aligned}$$

where $P_{\text {eff}}$ represents the actual driving force of coronary flow because $P^*_{\text {LV}}$ surrogates the effect of the pressure inside the left ventricular chamber. Since $P^*_{\text {LV}}$ is difficult to estimate, we introduce the following assumptions:

- In the ejection phase (from $T_0$ to $T_i$), pressure in the left ventricular chamber is slightly higher than $P_{\text {ar}}$ but its effects gradually decline moving from the endocardium towards the epicardium [24]. For this reason, we set $P^*_{\text {LV}} = 0.7 * P_{\text {ar}}$ as a global approximation.
- In the central phase of diastole (from the end of isovolumic relaxation up to the onset of the isovolumic contraction), ventricular pressure is negligible, so we set $P^*_{\text {LV}} = 0$.

During the isovolumic contraction and relaxation phases, $P_{\text {eff}}$ is modeled with $2{\text {nd}}$ order polynomials while ensuring continuity. In particular, during isovolumic relaxation (IR) we have:

$$\begin{aligned} {\left\{ \begin{array}{ll} P^{IR}(t) = a(t - T_{\text {notch}})^2 + P_{\text {notch}}, \\ P^{IR}(T_{i}) = 0.3*P_{\text {ar}}(T_i); \\ \end{array}\right. } \end{aligned}$$

whereas during isovolumic contraction (IC):

$$\begin{aligned} {\left\{ \begin{array}{ll} P^{IC}(t) = a(t - (T-IVCT))^2 + P_{\text {ar}}(T-IVCT), \\ P^{IC}(T) = 0.3*P_{\text {ar}}(T), \\ \end{array}\right. } \end{aligned}$$

where $IVCT = 0.05$ s is the isovolumic contraction time [21]. Summarizing, the effective pressure waveform $P_{\text {eff}}$ piecewise reconstructed with this method reads:

$$P_{\text {eff}}(t) = \left\{ \begin{array}{ll}0.3*P_{\text {ar}}&{}t\in [0, T_i], \\ P^{IR}&{}t\in [T_i, T_{\text {notch}}], \\ P_{\text {ar}}&{}t\in [T_{\text {notch}}, T - IVCT], \\ P^{IC}&{}t\in [T-IVCT, T]. \\ \end{array}\right.$$

From now on, the strategy to reconstruct the pressure curves $P_{\text {ar}}$ and $P_{\text {eff}}$ presented so far in “Pressure Waveform Reconstruction” and “Adaptation to Non-contracting Computational Models” sections will be referred to as *4-phases parametrization* technique.

### Data-Driven Estimation of Hyperemic Data

For the computational simulation of drug-induced hyperemic “stress” flow, the pressure waveforms obtained as in “Pressure Waveform Reconstruction” and “Adaptation to Non-contracting Computational Models” sections have to be reparametrized so that the effects of the drugs can be taken into account. To this aim, we used a database of 100 patients with direct measures of heart rate, systolic pressure, and diastolic pressure, before and after the administration of exogenous adenosine, to build regression lines to be used for the computation of the new stress parameters starting from their “rest” counterparts:

14 $$\begin{aligned} X_{\text {stress}} = \alpha \, X_{\text {rest}} +\beta . \end{aligned}$$

Being the database composed of clinical measures, a statistical elaboration based on interquartile range was performed first to spot and remove unreliable measures (statistical outliers). Setting $X_{\text {rest}}$ and $X_{\text {stress}}$ to be one of the measures of interest (heart rate or systolic/diastolic pressure) at rest and in stress conditions, respectively, the difference $\Delta X = X_{\text {rest}} - X_{\text {stress}}$ for each record was computed. Measure records were considered reliable if:

15 $$\begin{aligned}{}[Q_1 - 0.5*IQR]< \Delta X < [Q_3 + 0.5*IQR] \end{aligned}$$

with $Q_1$ and $Q_3$ being the first and third quartile of the distribution of $\Delta X$, respectively, and $IQR = Q_3 - Q_1$ is the interquartile range. Indeed, values of $\Delta X$ out of the range (13) the extremities of the distribution, likely representing unreliable measures. This dataset cleaning procedure ruled out 25 of the 100 records of the dataset.

### Coronary Blood Flow-Perfusion Model and Calibration

Hyperemic CBF-Perfusion simulations are ran using the computational model presented and used in [11, 12]. This model features a 3D description of blood fluid dynamics in the large arteries (Navier–Stokes equations, NS) and in the microcirculation (Darcy equations), suitably coupled through interface conditions representing mass conservation and forces balance.

NS is solved in a detailed coronary tree resulting from a single-vessel segmentation of cCTA images. This segmentation includes the 3 main coronary arteries (Left Anterior Descending, LAD, Left Circumflex, LCX, and Right Coronary Artery, RCA) alongside their major branches up to the imaging resolution limit (minimum diameter $D_{\text {min}} \simeq {0.8}\,\text {mm}$) and relies on the open-source software packages VMTK [25]. Segmentations were performed in a semi-automated way by the authors using a colliding fronts algorithm, with the supervision of an expert cardiologist. Segmentation parameters (e.g. lower and upper thresholds) were tuned dynamically so that the corresponding reconstruction matches the cCTA image. This calibration showed also good robustness with respect to the sensitivity of the results. To further improve geometrical accuracy, clinical evaluations of stenotic segments on cCTA images (in terms of stenosis degree) were also used as guidelines for the segmentations.

Blood dynamics in the small arteries and microcirculation is thought as a flow in a porous medium and accordingly solved with a homogenized Darcy problem. This is achieved through a multicompartment Darcy formulation that is solved in the left ventricle free wall [6], also segmented from rest cCTA images. Compared to other modeling solutions, for example those relying on lumped parameters at the coronary outlets, this 3D approach can quantify the spatial distribution of MBF at the tissue level and in every point of the muscle. Also, the porous homogeneization removes the need to solve flow equations in the whole coronary tree, which would be unfeasible due to the huge number of microvessels and ramifications.

We recall the three-compartment primal Darcy formulation used:

16 $$\begin{aligned} {\left\{ \begin{array}{ll} -\nabla \cdot (\varvec{K}_{1}^{-1}\nabla p_{M,1}) = g - \beta _{1,2}(p_{M,1} - p_{M,2}) \\ -\nabla \cdot (\varvec{K}_{2}^{-1}\nabla p_{M,2}) = \beta _{1,2}(p_{M,1} - p_{M,2}) - \beta _{2,3}(p_{M,2} - p_{M,3}) \\ -\nabla \cdot (\varvec{K}_{3}^{-1}\nabla p_{M,3}) = \beta _{2,3}(p_{M,2} - p_{M,3}) - \gamma (p_{M,3} - p_{\text {veins}}) \\ \end{array}\right. } \end{aligned}$$

where $p_{M,i}$ are the Darcy pressures of the *i*-th compartment, *g* is the mass source term in the first compartment coming from the NS equations, $\beta _{i,j}$ are the coefficients for mass exchange between compartments *i* and *j*, $\gamma$ is the venous drain and $p_{\text {veins}}$ is the (constant) venous pressure. Darcy compartments were defined as follows: 1-small arteries (mean diameter $\simeq 300 \quad \upmu m$), 2-arterioles (mean diameter $\simeq 75 \, \, \upmu m$), 3-capillaries (mean diameter $\simeq 5 \, \, \upmu m$). All the above parameters (except the source term *g* which comes from the solution of the NS problem) where here treated as constant in space and time. Table 4 reports the list of the values used for the parameters for all the patients.

**Table 4:** List of Darcy parameters used in the simulations. Permeabilities $K_i$ and venous pressure $p_{\text {veins}}$ were set according to previous studies [7]

Parameter	Value
$K_i$	$2 \times 10^{-9} \textrm{m}^{2} \textrm{Pa}^{-1} \textrm{s}^{-1}$
$\beta _{1,2}$	$2.5 \times 10^{-5} \textrm{Pa}^{-1} \textrm{s}^{-1}$
$\beta _{2,3}$	$1.25 \times 10^{-5} \textrm{Pa}^{-1} \textrm{s}^{-1}$
$\gamma$	$3 \times 10^{-5} \textrm{Pa}^{-1} \textrm{s}^{-1}$
$p_{\text {veins}}$	5 mmHg

Conductances $\beta _{i,j}$ and venous drain $\gamma$ were calibrated (see point 2 in the list of “Coronary Blood Flow-Perfusion Model and Calibration” section)

The new model setup with respect to the hyperemic state was performed at various levels:

1. Anatomic variations. Dilation of the large arteries was accounted through a uniform radial dilation by a factor of 1.225. This value was estimated using data regarding the diameter of distal RCA (of the eight patients at disposal) in both resting and stress conditions. The diameter measures were performed directly on the rest cCTA and stress CTP images by an experienced cardiologist. The dilation factor was then taken as the average, among all the patients, of the ratio between these stress and rest diameter measures.
2. Physiologic variations—myocardial parameters. Dilation of the microcirculatory vessels is taken into account through a calibration of the parameters of the Darcy model. Differently from what we did in [12], where we used data from the stress-CTP maps for this calibration, we here propose the following “blinded” approach. Conductances $\beta _{i,j}$ and venous drain $\gamma$ were first calibrated at rest to achieve two targets: firstly, a total arterial inflow of ${1}\,\textrm{mL}\,\textrm{min}^{-1} \textrm{g}^{-1}$ in patient P1, which has angiographically normal arteries. This choice was made to rule out eventual autoregulation mechanisms that may be present in pathological conditions. Secondly, the recovery of a pressure distribution, along the microvasculature (i.e. in the three compartments), in accordance with experimental measures [26]. Stress conductances were then obtained so that the total conductance would be 4 times its resting value, as suggested by previous findings [8] and to recover the changes in the pressure distribution along the microvasculature experimentally observed in the hyperemic state [26]. Importantly, the same parameters calibrated this way on patient P1 were used also for all the other patients P2–P8 and represent therefore the first viable approach towards a predictive application.

**Fig. 2:**
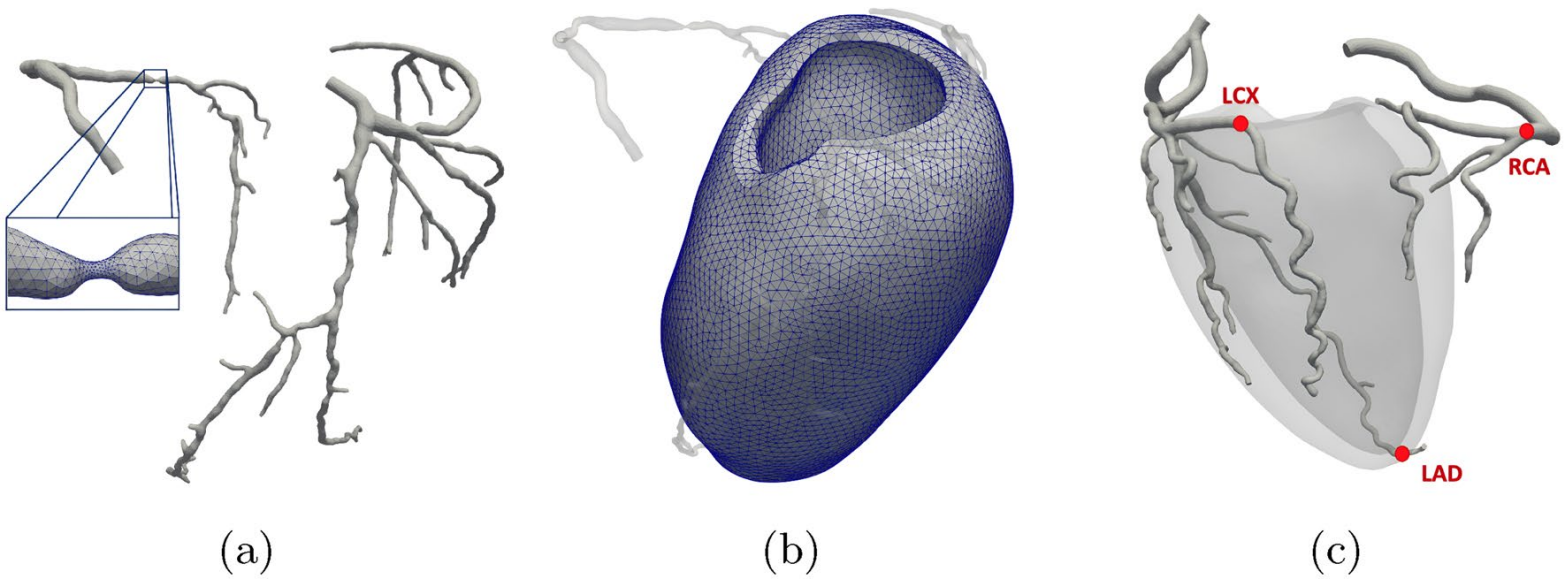
**a**, **b** Segmented domains and meshes for patient P7 used for the simulations of 3D blood fluid dynamics (**a**) and multicompartment Darcy (**b**) problems. **c** Landmarks on the coronary tree used for the computation of FFR values for patient P2 (shown as an example of left coronary dominance). Notice that the landmark for RCA is placed earlier than the interventricular and posterior branches, and the landmark for LCX is placed earlier than the posterolateral descent

The final geometry of the large coronaries was meshed with radius-dependent tetrahedral elements, while the left ventricle free wall was meshed with uniform hexahedral elements, as reported in Fig. 2 for patient P7.

Operational time for segmentation and geometry preparation was in the order of few hours per patient, while the model calibration, as highlighted at step 2 above, was performed *una tantum* on patient P1 and, thus, did not require any additional time for the other patients. Numerical treatment of the convective term in the Navier–Stokes equations, quantities related to blood rheology (Newtonian) and other numerical quantities (time step, tolerances, solution algorithms, preconditioners etc.) were the same as previously used in [12]. CBF-Perfusion simulations were run using the software life^x^, a high performance library for finite element simulations of multiphysics, multiscale and multidomain problems developed at MOX—Dipartimento di Matematica—Politecnico di Milano, within the iHEART project [27]. Simulations were run on the MOX—hpc cluster system for parallel computing, using processors Intel Xeon E5-2640v4 @ 2.40GHz, O.S. CentOS 7 with 56 cores; average simulation time was 3 h per patient. Thus, a complete analysis for each patient requires about 10 h including postprocessing of the results. Considering that this analysis is not required to run online (as in an interventional setting, for example), we believe that such time is compatible with this specific diagnostic application, where an answer is needed within a few days.

### Quantities for Validation

Hyperemic CBF-Perfusion simulations as described in “Coronary Blood Flow-Perfusion Model and Calibration” section were ran in eight patients. The selected patients were chosen among the patients enrolled in the PERFECTION clinical study [28], with no additional exclusion criteria other than the ones of the clinical study itself. A first subgroup of four patients was randomly selected among the patients scheduled for revascularization, whereas the second subgroup of four was randomly selected among patients whose treatment of choice was exclusively optimal medical therapy. For each of the eight patients, we have at disposal: contrast-enhanced cCTA acquisitions in resting conditions, ICA and invasive FFR measures, dynamic stress Computed Tomograhic Perfusion (stress-CTP) maps with quantitative information on myocardial blood flow (MBF) under stress conditions. Table 5 reports a summary of each patient’s clinical condition; for each of the above quantities, a suitable score is assigned.

**Table 5:** Clinical score of the patients population. Each score refers to the number of major coronary arteries (among LAD, LCX and RCA) having a positive outcome of the corresponding exam

Patient ID	ICA	FFR	stress-CTP
P1	0	0	0
P2	0	0	0
P3	1	0	0
P4	1	0	0
P5	3	2	3
P6	2	2	2
P7	3	3	3
P8	2	2	2

Score = n when: ICA - n coronaries features at least a lesion with % stenosis $> 70 \%$; FFR - n coronaries with FFR index $< 0.8$; stress-CTP - n perfusion territories (among the three principal ones) with MBF under stress < 150 ml/min/100 g

Post-processing of results and retrospective validation was performed using the outcomes of invasive FFR and stress-CTP. The FFR index in a point is formally defined as the ratio between the actual flow rate and the hypothetical flow rate that would occur in the same location in the absence of stenosis. In practice, FFR is approximated by the ratio between the mean of the measured pressure in that point and the mean aortic pressure. Accordingly, the in silico counterpart of the FFR index ($FFR_{\text {CT}}$) was computed over the whole coronary domain as:

17 $$\begin{aligned} FFR_{\text {CT}}(\varvec{x}) = \frac{P(\varvec{x})}{P_{\text {inlet}}}, \end{aligned}$$

which is an approximation valid at maximal hyperemia, where P is the computed blood pressure and $P_{\text {inlet}}$ is the pressure at the aortic root, which coincides with the prescribed boundary condition. The $FFR_{\text {CT}}$ values are computed at specific landmarks chosen according to the clinical methodology during invasive *FFR* measures and reported in Fig. 2c in the case of patient P2:

- LAD: distal segment, at the ventricle apex;
- LCX: distal segment prior to the posterior descending artery, in the case of left-dominant heart (i.e. when the posterior descending branch originates from the LCX); end of LCX, otherwise;
- RCA: distal segment prior to the bifurcation into the posterior and interventricular arteries.

Despite other indexes related to blood flow, which could be measured in resting conditions, have shown similar diagnostic performances compared to *FFR*, such as the Instantaneous Wave-Free Ratio (iFR) [29], the presence of autoregulation mechanisms reducing microvasculature resistance in response to a stenosis makes it very hard to predict the exact amount of resistance drop in resting conditions. Since this heavily impacts the MBF, such indexes were not computed by running simulations of resting flow. To ensure that the general hemodynamic conditions were actually representative of the patient’s state, we computed $MBF_{\text {comp}}$ as the average MBF (over the left ventricular volume and over the whole cardiac cycle) from the results of the simulations (see [12] for details on its definition) and then directly compared it to the $MBF_{\text {ctp}}$, taken as the in-space average MBF obtained from the perfusion maps given by the stress-CTP exam.

## Results

### Inlet Hyperemic Pressure Curves

**Fig. 3:**
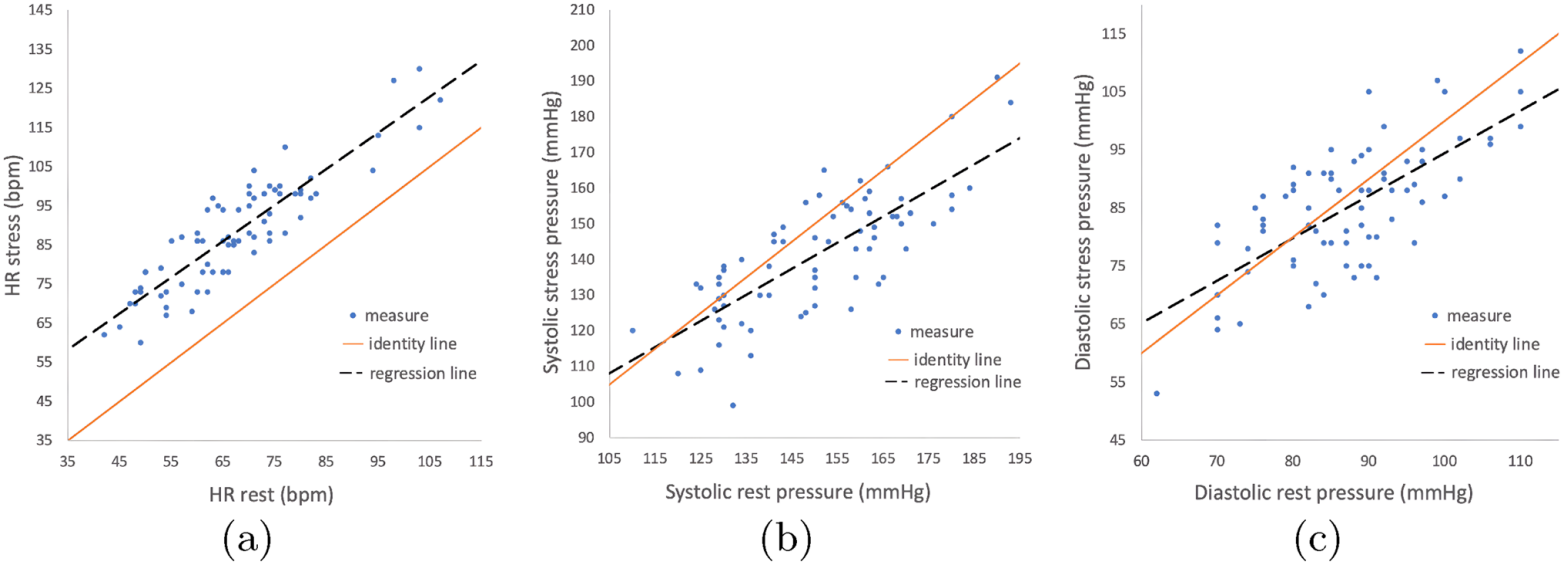
**a** Regression line built on clinical data of rest and stress heart rate measures. **b**, **c** Regression lines built on clinical data of rest and stress systolic/diastolic pressure measures

Figure 3 reports the regression lines to relate hyperemic and resting state quantities, obtained by applying the method described in “Data-Driven Estimation of Hyperemic Data” section to the clinical dataset at disposal which includes values of *HR*, systolic and diastolic pressures $P_{\text {sys}}$ and $P_{\text {dia}}$ (at rest and in stress conditions). In Table 6 we report, for all the three quantities of interest, the values of $\alpha$ and $\beta$ in (14) and the corresponding correlation coefficient *R*. The distributions of the data highlight two effects of adenosine: a relevant increase in heart rate and a slight decrease in both systolic and diastolic blood pressures.

**Fig. 4:**
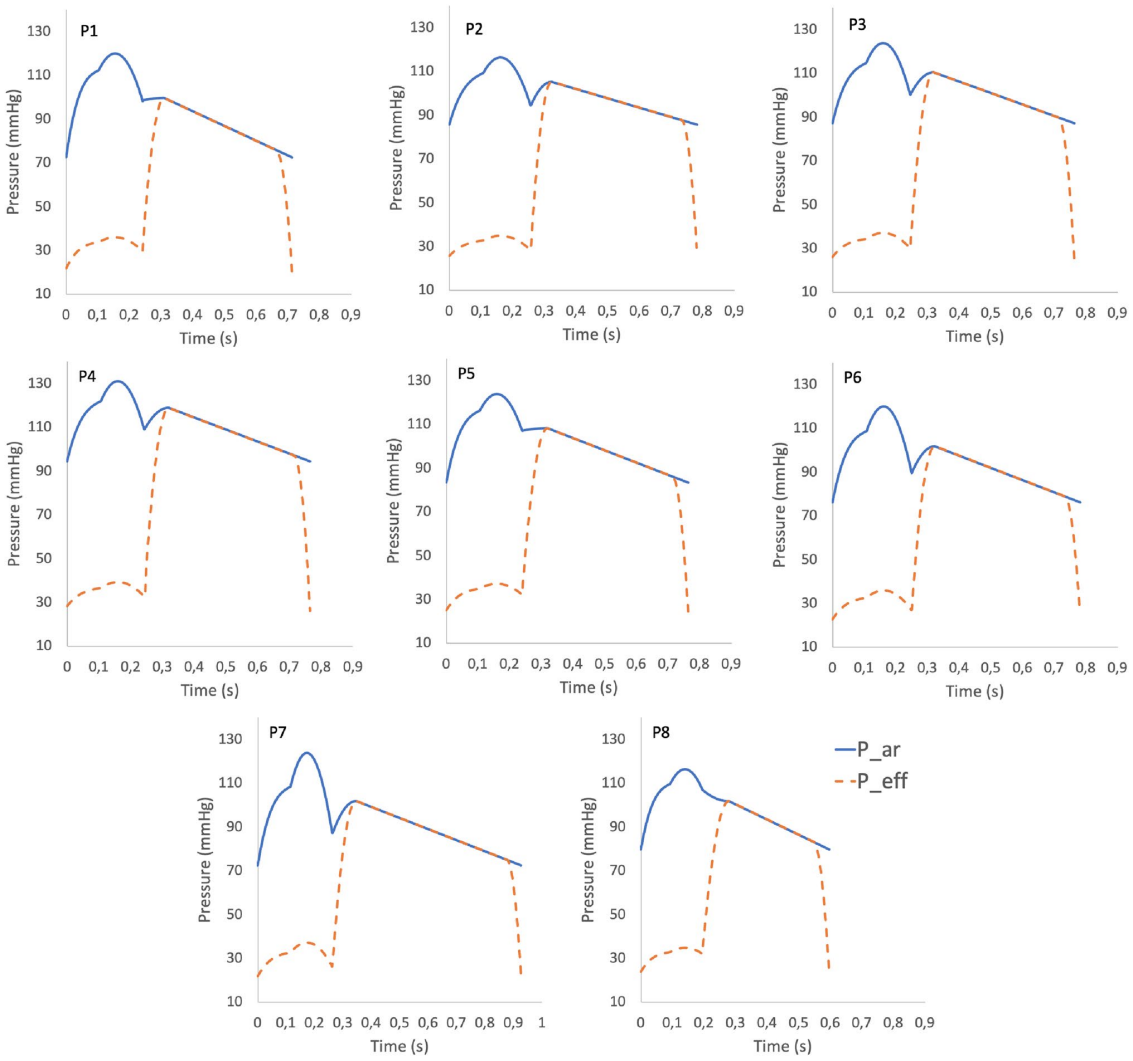
Aortic root $P_{\text {ar}}$ and effective pressure $P_{\text {eff}}$ curves reconstructed with the 4-phases parametrization technique described in “Methods” section for the patients P1–P8

Figure 4 reports, for the hyperemic case, the aortic root pressure $P_{\text {ar}}$ and the effective pressure $P_{\text {eff}}$ curves over a cardiac cycle for the patients P1–P8, obtained with the 4-phases parametrization technique described in “Methods” section.

From these results, we notice that all features characterizing the aortic pressure waveform were captured by our technique, which was able to recover physiological ranges and specific morphology of the curves. Notice also that, for construction, the two curves coincide during most of diastole. Instead, during systole, $P_{\text {eff}}$ is smaller as it is meant to surrogate the systolic impediment of flow due to the contraction.

**Table 6:** Coefficients and *R* values obtained with the rest-stress regression for the specific units of measure reported

Parameter	$\alpha$	$\beta$	*R*
*HR* bpm	0.92	26.00	0.89
$P_{\text {sys}}$ mmHg	0.73	31.10	0.78
$P_{\text {dia}}$ mmHg	0.73	21.25	0.70

### Hyperemic CBF-Perfusion Simulations

**Fig. 5:**
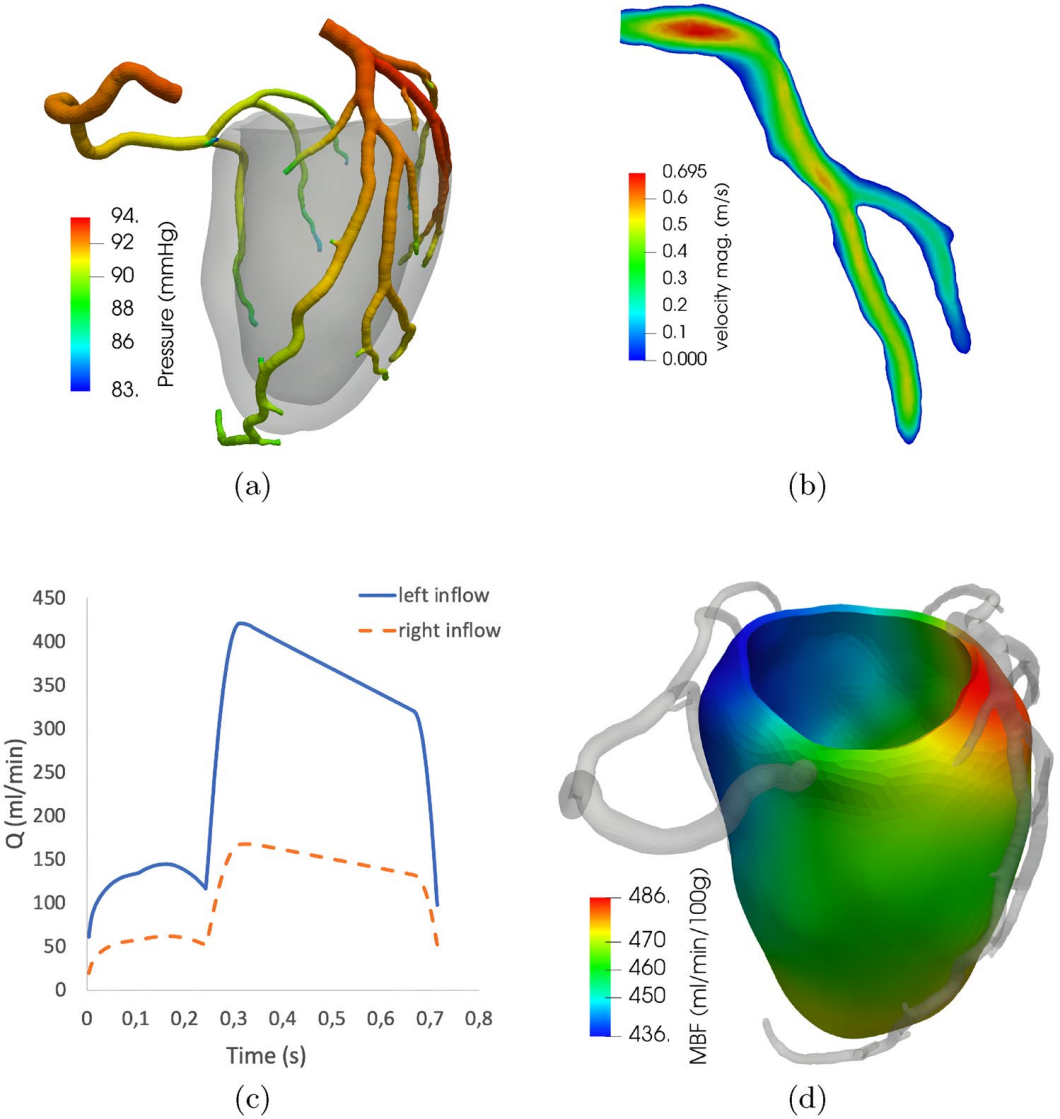
**a** Pressure field in the large coronaries computed at peak diastolic pressure ($t = 0.3 \,$ s). **b** Velocity field computed at mid diastole ($t = 0.5 \,$ s) in the LAD; the slicing plane is aligned with the LAD centerline in the middle segment but not at the inlet. **c** Left/right coronary blood flow *Q* computed over time at the left/right coronary inlets. **d** Istantaneous myocardial blood flow (MBF) in the left ventricle free wall computed at mid diastole ($t = 0.5 \,$ s). Patient P1

Figure 5 reports the computational results obtained, by means of the coupled CBF-Perfusion model, as described in “Coronary Blood Flow-Perfusion Model and Calibration” section, in the 3D coronary and myocardial domains for patient P1. In particular, we show the velocity and pressure fields in the large arteries and $MBF_{\text {comp}}$ in the left ventricle free wall, all computed in diastole. Notice that physiological pressure, pressure gradient, and velocity magnitude are recovered along all the coronary branches. In order to highlight the recovery of the characteristic diastolic flow, we also report the computed flow rate *Q* at the coronary inlets. We can notice that the right coronary flow is substantially lower than the left one even if the flow subdivision has not been prescribed a priori. This means that the flow subdivision is regulated solely by the anatomy of the coronary tree and that our proposal for the inlet pressure condition can be used without any additional assumption.

**Fig. 6:**
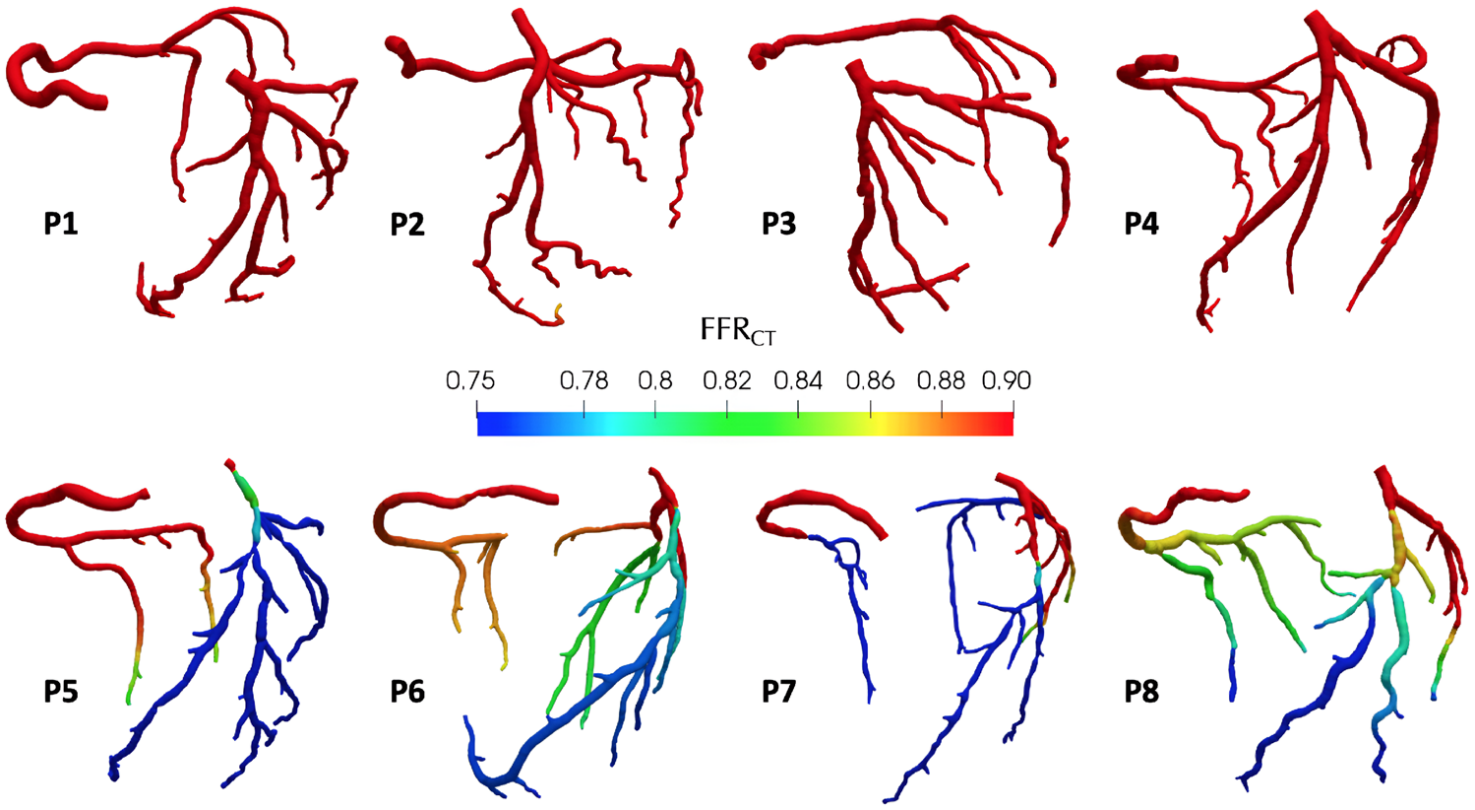
$FFR_{\text {CT}}$ results for patients P1–P8, computed over the whole coronary domain. Patients 5–8 have at least one major artery with positive outcome of the invasive *FFR* exam (invasive $FFR \, < \, 0.8$)

Figure 6 reports, starting from the computational solution of the CBF-Perfusion model, the $FFR_{\text {CT}}$ values for patients P1–P8, computed by means of (17), at the (patient-dependent) time corresponding to the peak diastolic pressure. $FFR_{\text {CT}}$ results showed little variation in time during the diastolic phase, so we do not expect different results at other time instants. These results allow us to reproduce with great accuracy the outcomes of the invasive *FFR* procedure, leading to an effective identification of functionally significant lesions characterized by $FFR < 0.8$.

**Table 7:** FFR results: quantitative comparison in the form: $FFR_{CT}$—invasive *FFR*, at the specific landmarks reported in Fig. 2c, of each major coronary artery

Patient ID	LAD	LCX	RCA
P1	0.95 – 0.9	0.93 – 0.9	0.96 – 0.9
P2	0.88 – N	0.92 – N	0.97 – N
P3	0.92 – 0.88	0.96 – 0.96	0.96 – 0.9
P4	0.93 – 0.9	0.96 – 0.9	0.97 – 0.95
P5	0.62 – 0.5	0.65 – 0.5	0.92 – 0.84
P6	0.73 – 0.5	0.82 – 0.5	0.89 – 0.91
P7	0.53 – 0.5	0.61 – 0.5	0.48 – 0.5
P8	0.71 – P	0.91 – N	0.84 – N

According to clinical practice, in the case of mild ($< 30 \%$) or critical ($> 90 \%$) stenoses the invasive measure in general is not performed and the score N (negative) or P (positive) is instead assigned, respectively

In Table 7, the quantitative comparison between $FFR_{CT}$ values and invasive *FFR* measures, for each major artery of each patient, is reported. According to the clinical methodology, in the case of mild ($< 30 \%$) and critical ($> 90 \%$) stenosis the invasive *FFR* measure is not performed, since in such conditions FFR is supposed to be large ($> 0.8$) and small, respectively. Accordingly, in such cases a negative (N) and positive (P) score is directly assigned by clinicians. In the other cases, we report the exact invasive *FFR* measure. We notice the excellent agreement between *FFR* predictions and invasive measures.

**Fig. 7:**
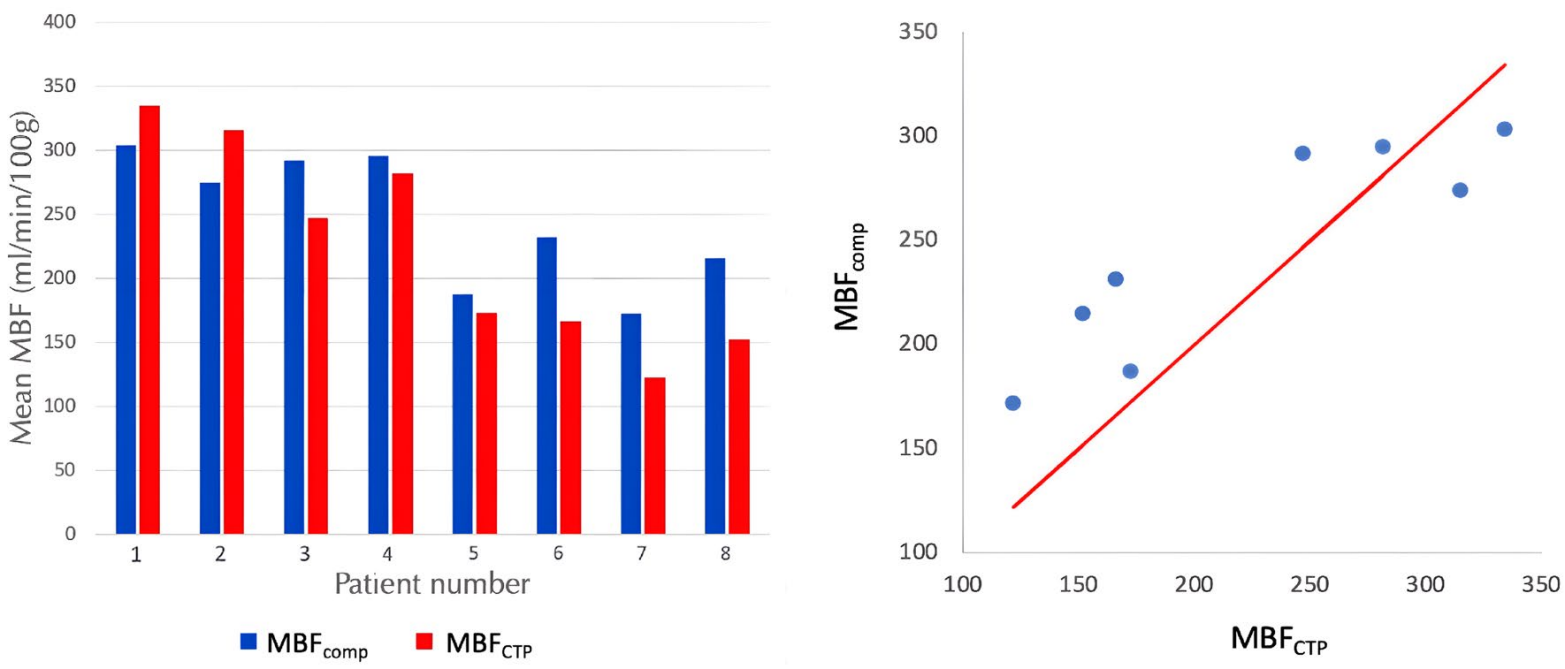
Quantitative comparison between computed $MBF_{\text {comp}}$ vs clinical $MBF_{\text {ctp}}$ values of myocardial blood flow in patients 1–8. Patients 4–8 have at least a principal perfusion territory with positive outcome of the stress-CTP exam ($MBF_{\text {ctp}} \, < 150 \,$ ml/min/100g).

Figure 7 reports the mean value of MBF for patients P1–P8, both in space over the whole myocardium and in time over the whole cardiac cycle, compared with the values extracted from the stress-CTP maps. We notice that, in all the patients, the average $MBF_{\text {comp}}$ is always in the physiological range for stress conditions.

## Discussion

In the context of prognostic stratification of Coronary Artery Disease, the assessment of coronary flow and myocardial perfusion in an hyperemic state is of crucial importance. Maximal hyperemia leads to the exhaustion of the autoregulation mechanisms of the coronary circulation, which in the case of severe pathological conditions may not be able to keep up with the augmented metabolic demand of the cardiac muscle, resulting in major adverse events. Indeed, several clinical studies have shown a high predictive value of *FFR* and hyperemic MBF with respect to the most commonly used clinical endpoints (major adverse event, need for hospitalization/surgical intervention) [2, 3, 5].

Within the context of predictive computational analysis of hyperemic flow, the prescription of accurate inlet conditions at the coronary ostia is a daunting task. Direct measures of coronary inflow at rest are usually not available and, even when they are, its knowledge does not allow to estimate the inflow in the hyperemic state. Using a constant pressure inlet condition, for example the mean arterial pressure, is also not ideal because of the well-known flow impeding effect that occurs in systole due to cardiac contraction. Instead, the prescription of a pressure waveform over time would allow the modeling of the effects of cardiac contraction as a surrogate and, thus, represents an easy and effective solution. Nonetheless, this choice introduces many challenges, namely building the full waveform over time at the coronary ostia starting from instantaneous brachial measures and the estimation of the modifications occurring in the hyperemic state. As alternative strategies, one may consider a time-varying microvascular resistance given by a mathematical expression, see e.g. [30, 31], or the application of an external pressure (mimicking the pressure generated by contraction) to lumped parameter models representing the peripheral circulation [32]. Notice that, since we wanted to compute the 3D distribution of MBF, the downstream boundary condition for the coronary flow is provided by a 3D multi-compartment Darcy model. In this context, the implementation of the above mentioned alternative strategies would require an extension and adaptation, which would deserve further investigation.

In this work, we propose a novel strategy to build, along the whole heartbeat, the pressure waveform at the aortic root in the two physiological states of rest and hyperemia. To describe the physiological behaviour of such waveform, we considered a piecewise building accounting for the different phases of the heartbeat. Notice that other works relied on the building of an inlet pressure curve to be prescribed at the inlet of a blood dynamics problem based on a piecewise mathematical representation. For example, in [33] the authors proposed a pressure curve built as piecewise solution of a suitable windkessel model.

Notably, our strategy needs only readily available clinical measures of heart rate and maximum/minimum brachial pressure at rest. Other works used instead aortic flow rate measures to build pressure curves, see for example [34].

In this context, recently proposed approaches based on Artificial Intelligence (AI) showed promising results in terms of robustness and handling of new data [35, 36] However, these methods are still hampered in their applicability by the lack of large and organized datasets (forcing the use of virtual datasets) and they usually require, as inputs, the full pressure signal over time recorded at the arm level. This is not always feasible in a clinical setting, where only the maximum an minimum pressure are typically measured. Nonetheless, the exploration of AI approaches, potentially combined with more established strategies as the one we proposed, could provide a very robust alternative in the future.

The use of our waveforms as inlet boundary conditions for CBF-Perfusion simulations, together with a novel model calibration strategy that does not rely on any prior knowledge of hyperemic flow, allowed us to obtain clinically meaningful results in eight patients, showing a high predictive power with respect to the *FFR* index and MBF. This is a major step forward compared to out previous works [11, 12], where both the inlet condition (based on hyperemic flow over time) and Darcy parameters were obtained using the stress-CTP map of the patient, which is one of the exams that this methods aims at predicting.

Our computational results show that the proposed framework is able to reproduce all the characteristic features of the coronary circulation: a physiological pressure gradient along the major arteries (a), mean velocities consistent with previous, direct measurements of coronary flow [37] (b), a mostly diastolic rather than systolic flow over the cardiac cycle (c) and a reasonable MBF distribution, with local heterogeneities in accordance with the direct observations for non-ischemic myocardium [38], showing the highest local MBF values in the anterolateral wall and the lowest in the posterior wall. As it can be seen in Fig. 5c, the characteristic diastolic flow is obtained also at the right coronary inlet, where it is known that systolic and diastolic flows are rather similar [37]. This is due to the presence, in the RCA, of side branches perfusing the right ventricle, that generates significantly lower pressures (compared to the left) and thus shows a much lower systolic impediment. As a consequence, in these branches systolic flow is preserved and so the total systolic flow at the right inlet is higher. Because we did not include such branches in our model, this feature was not recovered by the simulations. The definition of $P_{\text {eff}}$ proposed in (13) would need to be refined to take this effect into account. However, we point out that this effect does not have any impact on MBF in the left ventricle, which is largely the most clinically relevant.

From the $FFR_{\text {CT}}$ results reported in Fig. 6 and Table 7, we can see that our approach shows a high predictive power when applied to the patient-specific calculation of the *FFR* index for each major artery of each patient, successfully identifying functionally relevant lesions. Clustering the results following the clinical threshold of functional relevance when $FFR < 0.8$, we obtained a per-vessel sensitivity of 95.8% and specificity of 100%. The mean relative error computed from Table 7 was $13.7 \%$, although such quantitative comparison is based on a rather small number of vessels so validation with a larger database of patients would further improve the consistency of these results.

Most importantly, from the results of Fig. 7 we see that we could achieve a good accordance also for the mean MBF, especially in the non-ischemic cases (patients 1–4) where we report a mean error of $11.3 \%$. This is especially relevant because no assumptions or data are provided to the model regarding the flow. For patients 5–8, where perfusion defects were present, we see a slight yet systematic overestimation in MBF, with a mean error of $32.6 \%$. We hypothesize that this could be caused by two reasons: (a) an underestimation of stenosis severity in the phase of geometry reconstruction, (b) an underestimation of the effect of ventricular hypertrophy on diastolic flow. Patients 5, 7 and 8 showed varying degrees of left ventricular hypertrophy, which has been found to hamper diastolic flow due to the rarefaction of blood vessels in the thickened muscle [39] and the augmented compression forces on the microvasculature also in diastole [40]. Since this second effect is not included in our model, we believe that it could be the cause of the observed MBF overestimation for these patients. For patient 6, without ventricular hypertrophy, the most likely reason is an underestimation of stenosis severity, given also the relatively high values of $FFR_{CT}$ for this patient.

Previous works in computational modeling of coronary blood flow and myocardial perfusion have focused mainly on single aspects such as *FFR* computation [8, 41] or the recovery of physiological flow patterns in the microcirculation through complex poroelastic models [6, 42]. This work is one of the first attempts to run a computational analysis of coronary blood flow, on real patients data with various pathologies, with a clear predictive purpose with respect to both FFR index and MBF, representing an important step towards an integrated analysis of blood flow at all levels of the coronary circulation. To the best of our knowledge, it is also the first time that such analysis is run using a full pressure waveform as inlet condition in the hyperemic state. Compared to other solutions (e.g. the use of a constant pressure [7]), this is essential for an application to models explicitly including cardiac mechanics, and opens up interesting possibilities for the investigation of the effects on MBF of specific pathologies such as ventricular hypertrophy, aortic valve stenosis, augmented arterial stiffness and, in general, every condition that may alter the shape of the pressure waveform without significantly affecting its mean value.

To achieve a successful clinical application of this method, many challenges are still to be overcome. Firstly, an extended analysis on a larger number of patients is required, including also a stricter validation protocol with quantitative comparison on *FFR* at precise locations. Secondly, we notice that values of *FFR* close to the clinical threshold value of 0.8 are particularly critical to estimate, since even small errors in the simulation results (due for example to possible errors in geometry reconstruction) can lead to stenosis misclassifications. In this context, a higher number of vessels with invasive *FFR* close to the threshold value of 0.8 would provide a very solid benchmark. Thirdly, segmentation of the large arteries is a critical step in terms of accuracy (*FFR* is very sensitive to geometric inaccuracies) and operational time. Our approach is based on semi-automated strategies that are susceptible to inter-operator variability, requiring a good level of user expertise and significant operational time (in the order of few hours per patient). Automated and standardized segmentation strategies are therefore highly desirable. Compared to already established approaches in the field such as the HeartFlow analysis [8], our approach has the major advantage of expanding the simulation to the blood flow at the tissue level, which holds a high prognostic power, as demonstrated by recent clinical studies [28]. We believe that the tools proposed in this work represent a crucial step towards an integrated and predictive analysis of blood flow both at the level of the large arteries and in its distribution in the microcirculation.

The study presented in this work has some methodological limitations:

- The dispersion in the clinical data regarding rest-stress pressure values is rather high. A covariate analysis with more clinical parameters may improve the consistency of the method;
- Although different clinical conditions were included in the patients population, more cases are required to improve the consistency of the results;
- A direct validation, using invasively measured pressure recordings, would provide a solid benchmark for the absolute accuracy of the proposed strategy.
